# Supplementary material for: Gender and socio-economic stratification of ultra-processed and deep-fried food consumption among rural adolescents: A cross-sectional study from Bangladesh
Source: PLoS One. 2022 Jul 28;17(7):e0272275. doi: 10.1371/journal.pone.0272275 (PMC9333446; doi:10.1371/journal.pone.0272275)
Supplement: S1 Table — (DOCX) [file pone.0272275.s001.docx]

Supporting information

**Table S1.** Food items belonging to the four groups of ultra-processed foods and one group of deep-fried foods for which consumption was assessed during the household survey.

| **Food groups** | **Food items in the group** |
| --- | --- |
| **Ready-to-eat or “instant” foods (UPF)** | Instant noodles (Maggi noodles), burgers, industrially mass-produced breads/loaves and buns. |
| **Confectionery, sweets, and similar packaged products (UPF)** | Biscuits or cookies, chocolates, candies; industrially mass-produced, packaged muffins and cakes, ice cream |
| **Savory snacks (UPF)** | Potato chips, crisps, *chanachur*, other salty/spicy packaged snacks, such as roasted peanuts, *jhalmuri*. |
| **Sugar-sweetened beverages (UPF)** | Soft drinks (Coca-Cola, Pepsi, Mirinda, Fanta), *Jeera Pani* (bottled, sweetened cumin water), energy drinks (Tiger, Shark, Speed, Power, etcetera), mix-and-drink sachet (such as Tang). |
| **Deep-fried foods** | *Shingara*, samosa, *puri, fuchka,* lentil fritters, french fries. |

UPF: ultra-processed food; Bengali names are italicized.
