# Supplementary material for: Gender and socio-economic stratification of ultra-processed and deep-fried food consumption among rural adolescents: A cross-sectional study from Bangladesh
Source: PLoS One. 2022 Jul 28;17(7):e0272275. doi: 10.1371/journal.pone.0272275 (PMC9333446; doi:10.1371/journal.pone.0272275)
Supplement: S2 Table — (DOCX) [file pone.0272275.s002.docx]

**Table S2.** Logistic regression analyses of association between socio-demographic variables and consumption of ultra-processed and deep-fried foods.

| **Variables** | **Ready-to-eat or “instant” foods** | | **Confectioneries, sweets, similar packaged products** | | **Savory snacks** | | **Sugar-sweetened beverage** | | **Deep-fried food** | |
| --- | --- | --- | --- | --- | --- | --- | --- | --- | --- | --- |
|  | **Crude OR**  **(95% CI)** | **Adjusted OR^1^**  **(95% CI)** | **Crude OR**  **(95% CI)** | **Adjusted OR^1^**  **(95% CI)** | **Crude OR**  **(95% CI)** | **Adjusted OR^1^**  **(95% CI)** | **Crude OR**  **(95% CI)** | **Adjusted OR^1^**  **(95% CI)** | **Crude OR**  **(95% CI)** | **Adjusted OR^1^**  **(95% CI)** |
| **Gender:**  Girl (Ref.) |  |  |  |  |  |  |  |  |  |  |
| Boy | 1.96  (1.54-2.50)* | 1.85  (1.45-2.38)* | 1.22  (1.04-1.43)* | 1.22  (1.04-1.44)* | 1.20  (1.02-1.41)* | 1.19  (1.01-1.41)* | 2.72  (2.10-3.55)* | 2.57  (1.97-3.37)* | 1.97  (1.67-2.32)* | 1.96  (1.66-2.32)* |
| **Wealth:**  Poorest (Ref.) |  |  |  |  |  |  |  |  |  |  |
| Middle-status | 1.24  (0.92- 1.67) | 1.25  (0.92-1.72) | 1.12  (0.92-1.36) | 1.08  (0.89-1.33) | 0.91  (0.74-1.11) | 0.91  (0.74-1.13) | 1.25  (0.92-1.71) | 1.21  (0.88-1.68) | 1.09  (0.89-1.33) | 1.08  (0.88-1.33) |
| Richest | 1.46  (1.09-1.96) | 1.55  (1.12-2.16)* | 1.26  (1.04-1.53)* | 1.19  (0.96-1.48) | 1.13  (0.93-1.39) | 1.17  (0.93-1.46) | 1.43  (1.06-1.95)* | 1.44  (1.02-2.03)* | 1.16  (0.95-1.41) | 1.21  (0.97-1.51) |
| **Maternal education:**  No education (Ref.) |  |  |  |  |  |  |  |  |  |  |
| Primary | 1.00  (0.72-1.40) | 0.96  (0.68-1.35) | 0.94  (0.76-1.18) | 0.91  (0.72-1.14) | 0.95  (0.75-1.20) | 0.94  (0.75-1.19) | 1.14  (0.81-1.63) | 1.13  (0.79-1.63) | 0.93  (0.75-1.17) | 0.92  (0.73-1.16) |
| Secondary and above | 0.99  (0.73-1.38) | 0.85  (0.60-1.21) | 1.08  (0.87-1.33) | 0.96  (0.76-1.22) | 0.95  (0.76-1.19) | 0.89  (0.70-1.14) | 1.12  (0.81-1.58) | 0.99  (0.69-1.45) | 0.87  (0.70-1.08) | 0.79  (0.62-1.00) |
| **Adolescents´ education:**  Primary and below (Ref.) |  |  |  |  |  |  |  |  |  |  |
| Secondary | 0.72  (0.55-0.96)* | 0.78  (0.58-1.04) | 1.12  (0.92-1.36) | 1.13  (0.92-1.39) | 0.94  (0.77-1.16) | 0.97  (0.79-1.21) | 0.65  (0.49-0.87)* | 0.73  (0.54-0.98)* | 0.85  (0.70-1.04) | 0.99  (0.80-1.22) |

OR: odds ratio; CI: confidence interval. ^1^Adjusted for gender, household wealth, maternal and adolescents´ education simultaneously. *Asterisk indicates statistical significance as the CI excludes 1.
