# Supplementary material for: Gender and socio-economic stratification of ultra-processed and deep-fried food consumption among rural adolescents: A cross-sectional study from Bangladesh
Source: PLoS One. 2022 Jul 28;17(7):e0272275. doi: 10.1371/journal.pone.0272275 (PMC9333446; doi:10.1371/journal.pone.0272275)
Supplement: Attachment — Submitted filename: Response to Reviewers.docx [file pone.0272275.s004.docx]

Dear Editor,

We would like to extend our appreciation for the constructive inputs received from the learned reviewers. We present our responses to the comments and revisions made herewith. Please note that the line numbers mentioned are those of the revised manuscript with track changes.

**Reviewer #1**

Abstract

Avoid abbreviation from abstract

**Response**

*We have omitted the abbreviation for sugar-sweetened beverage (i.e., SSB) and used the full form throughout the Abstract. (Lines 32, 39–40, 41 and 46)*

Introduction part

First start with the definition of your outcome variable clearly

**Response**

*We have revised accordingly and added the following at the beginning of the Introduction: “Ultra-processed foods (UPFs) refer to multi-ingredient, industrial formulations composed of such food-derived substances as starch, fat, oil, sugar, casein, etcetera; or synthesized through complex processing of food constituents like whey, gluten, soya protein isolate, maltodextrin, and corn syrup [1,2]”. (Lines 53–56)*

When you write the magnitude of the problem be specific on your study population or among adolescents “It accounted for an estimated 11 million deaths and 53 255 million disability-adjusted life-years (DALYs) in 2017” this sentence is not clear among adolescent or the general population?

**Response**

*We have added “among adults” for clarification. (Line 58)*

The second paragraph is very long from L#58 –L#81 divided into two or three paragraphs to make easy for reader

**Response**

*As suggested, we have divided this paragraph into two shorter paragraphs: one dealing with the increasing consumption of ultra-processed foods (Lines 63–76), and another dealing with the problem posed by deep-fried foods (Lines 77–86).*

In the last paragraph the gap you mentioned not clear?

**Response**

*In the last paragraph of the Introduction, we highlighted the issue that adolescents in low- and middle-income countries (LMICs) now have a greater access to inexpensive calories, while the quality of their diet remains limited (Lines 88–97). In parallel to this, the trend of overweight and obesity among adolescents has accelerated in South Asian LMICs, but the trend remained stable in high-income countries (Lines 97–98). This observation is reported in a large, pooled analysis by Abarca-Gómez and colleagues (manuscript Reference number 35) that was published in The Lancet in 2017 [1]. Thereafter, we pointed out that adolescents in Bangladesh are also facing a similar situation (Lines 98–100). Consumption of ultra-processed and deep-fried foods is a well known driver of the rising burden of overweight and obesity. However, empirical evidence on gender and socio-economic stratification of consumption of these unhealthy foods among Bangladeshi adolescents is lacking. This is the “gap” that the present study aimed to address. Context-specific understanding of the relationship of gender and socio-economic factors with consumption of these unhealthy foods is necessary for informing targeted public health initiatives.*

*For clarity, we revised as follows: “... Ultra-processed and deep-fried foods are known dietary drivers of increasing overweight and obesity [15,18]. Nevertheless, socio-demographic correlates of consumption of these unhealthy foods among rural, Bangladeshi adolescents remain unexplored. Hence, we aimed to describe ultra-processed and deep-fried food consumption among rural adolescents by gender and socio-economic factors, and to explore their relative importance in shaping consumption.”. (Lines 100–106)*

Methods

In L# 137 -138 “Reported consumption of roughly one tablespoonful or more of one or more items qualified the participant as consumer of that particular group of ultra-processed or deep-fried food.” Is there any reference?

**Response**

*This requirement of consumption of roughly one tablespoonful was intended for avoiding foods eaten in trivial amounts from categorizing a participant as a consumer. This has been recommended in the context of dietary assessment using food groups [2], and employed in our previous work in Matlab [3] as well.*

*We have now added the reference to the manuscript (Manuscript Reference number 41). (Line 145).*

Result

In table 1 for categorical variable (for example gender, educational status, source of drinking water) why you report mean and SD better to report frequency and percentage?

**Response**

*As suggested, we reported frequency with percentage for categorical variables and mean with standard deviation for numerical variables. The heading of the right column of Table 1 reads “n (%) or mean (SD)”, which indicates that for categorical variables like gender, educational status, etcetera the values represent frequency (“n” stands for frequency here) along with percentage in the parentheses. For example, in case of gender, we had 1261 girls (the frequency or n) that constitute 51.2% (the percentage in the parentheses) of the study sample. Therefore, the value 51.2 in the parentheses does not represent standard deviation, rather represents the percentage of girls in the sample.*

*We have added a legend to the Table 1: “The values represent frequency (n) with percentage for categorical variables and mean with standard deviation (SD) for numerical variables”. (Lines 197–198)*

Discussion part

Your discussion part lack coherence, so, need revision

Better to start the discussion with your objective then explain your result with the first paragraph

**Response**

*We have added the following at the beginning of the Discussion: “The objective of the present study was to describe and analyze the gender and socio-economic stratification of ultra-processed and deep-fried food consumption in a cohort of rural adolescents”. (Lines 242–244)*

Then compare your finding with other study findings like consistence with, higher or lower than after that for the difference write possible explanation and clinical implication of your finding

**Response**

*Regarding the structure of the Discussion section: we presented an abridged summary of the principal findings in the first paragraph (Lines 242–252).*

*In the second paragraph (Lines 253–277), we focused on the extent of consumption in terms of the proportion of adolescents who reported to have consumed ultra-processed and deep-fried foods. We contextualized the extent of consumption with regard to our study setting, which is a rural area (Lines 253–264). We, then, focused on the reported consumption of deep-fried foods which was fairly high and discussed the broader implications of deep-fried food consumption (Lines 264–277). Overall, this paragraph puts into perspective the described consumption of the five groups of unhealthy foods.*

*In the third paragraph (278–291), we focused particularly on sugar-sweetened beverage and read-to-eat or “instant” foods. Consumption of these two groups were relatively low in our sample, and thus, contrasted with findings from urban settings elsewhere (Lines 279–285). Furthermore, these are the two food groups (out of five) for which household wealth showed a positive association with consumption (Table 3 in the manuscript). Therefore, we suggested that this could indicate a potential role of purchasing ability (Lines 283–286). In the last two sentences of this paragraph, we compared our findings regarding household wealth status (i.e., positive association with consumption for only two food groups) with that of previous studies (Lines 289–291).*

*In the fourth paragraph (292–308), we offered a context-specific perspective on the emerging problem of ultra-processed food consumption in the rural setting of Matlab based on our previous research in the area. We have also added a brief discussion on clinical implication as suggested by the Reviewer (Lines 301–308, please see below for further clarification).*

*In the fifth paragraph (Lines 302–317), we highlighted the gender dimension in our findings – one of the main focuses of the study. Furthermore, we compared and contrasted the gender stratification observed in our study with that of the existing studies from elsewhere.*

*In the last paragraph (Lines 318–328), we mentioned pertinent limitations and methodological considerations.*

*We perceive that the overall structure of the Discussion section serves the purpose of highlighting the major findings in line with the stated aim of the study and contextualizing them against the backdrop of what has been reported in existing studies.*

*Discussing clinical implications of consuming ultra-processed and deep-fried foods within the scope of a cross-sectional study among adolescents as clinical outcomes manifest often in ensuing adulthood. However, in line with the suggestion, we added the following: “Consumption of ultra-processed and deep-fried foods has been associated with several clinical outcomes including metabolic syndrome, hypertension, dyslipidemia and coronary artery disease [25,61]. These associations were observed predominantly among adults, yet the underlying cardiometabolic alterations may commence during adolescence [62]. Besides, the habit of consuming unhealthy foods established during adolescence can track into adulthood [63]. Accordingly, the gender stratification observed in this study might play a role in exposing the boys to a higher cardiometabolic risk in upcoming adulthood”. (Lines 301–308)*

Why you discuss the significantly associated factors of UPF and deep fired food? For example gender, wealth quintile

**Response**

*We highlighted significantly associated factors in the Discussion for two reasons: (i) their direct relevance to the stated aim of the study, which is “Hence, we aimed to describe ultra-processed and deep-fried food consumption among rural adolescents by gender and socio-economic factors, and to explore their relative importance in shaping consumption” (Lines 104–106); and (ii) it would be erroneous to draw inference from results (particularly odds ratios) that were not statistically significant.*

*Our aim was to investigate the gender and socio-economic stratification. We found that gender was significantly associated with consumption for all five food groups whereby the odds of consumption were significantly higher among boys than girls. On the other hand, odds of consumption were significantly higher among adolescents from the richest households only for two food groups: ready-to-eat or “instant” foods and sugar-sweetened beverage. We drew inferences accordingly.*

In the conclusion part: conclude based on your finding for example ‘Household wealth was associated with the consumption of ready-to-eat or “instant” foods 222 and SSB”

**Response**

*As suggested, we have added the following: “Household wealth was associated with consumption of ready-to-eat or “instant” foods and SSB, but not the other three food groups. This may indicate a role of purchasing ability in the rural setting” in the Conclusion section. (Lines 342–344)*

**Reviewer #3**

The paper focuses on very interesting topic, very important segment of population (Adolescents) and uses large datasets. I am happy on this and I personal recommend it for acceptance of publication. As a comment, please try to address some grammatical, editorial issues, and in some part; clarity of some statements (for easily understandability for readers) will be checked and addressed.

**Response**

*We greatly appreciate the comments from the Reviewer. We have revised and made adjustments for further clarity. (Lines 100–104, 242–244, 301–308).*

**References**

1.	Abarca-Gómez L, Abdeen ZA, Hamid ZA, Abu-Rmeileh NM, Acosta-Cazares B, Acuin C, et al. Worldwide trends in body-mass index, underweight, overweight, and obesity from 1975 to 2016: a pooled analysis of 2416 population-based measurement studies in 128·9 million children, adolescents, and adults. The Lancet. 2017;390: 2627–2642. doi:10.1016/S0140-6736(17)32129-3

2.	Arimond M, Wiesmann D, Becquey E, Carriquiry A, Daniels MC, Deitchler M, et al. Simple Food Group Diversity Indicators Predict Micronutrient Adequacy of Women’s Diets in 5 Diverse, Resource-Poor Settings. J Nutr. 2010;140: 2059S-2069S. doi:10.3945/jn.110.123414

3.	Islam MR, Rahman SM, Tarafder C, Rahman MdM, Rahman A, Ekström E-C. Exploring Rural Adolescents’ Dietary Diversity and Its Socioeconomic Correlates: A Cross-Sectional Study from Matlab, Bangladesh. Nutrients. 2020;12: 2230. doi:10.3390/nu12082230
